# Differences in Bacterial Co-Occurrence Networks and Ecological Niches at the Surface Sediments and Bottom Seawater in the Haima Cold Seep

**DOI:** 10.3390/microorganisms11123001

**Published:** 2023-12-18

**Authors:** Song Zhong, Jingchun Feng, Jie Kong, Yongji Huang, Xiao Chen, Si Zhang

**Affiliations:** 1 Guangdong Provincial Key Laboratory of Water Quality Improvement and Ecological Restoration for Watersheds, Institute of Environmental and Ecological Engineering, Guangdong University of Technology, Guangzhou 510006, China; 1112124003@mail2.gdut.edu.cn; 2 Research Centre of Ecology & Environment for Coastal Area and Deep Sea, Guangdong University of Technology, Guangzhou 510006, China; kongjie@gmlab.ac.cn (J.K.); yjhuang@scsio.ac.cn (Y.H.); chen_xiao@gmlab.ac.an (X.C.); zhsimd@scsio.ac.cn (S.Z.); 3 Southern Marine Science and Engineering Guangdong Laboratory (Guangzhou), Guangzhou 511458, China

**Keywords:** methane seepage, bacterial communities, spatial distribution, co-occurrence networks, community construction, surface sediments

## Abstract

Cold seeps are highly productive chemosynthetic ecosystems in the deep-sea environment. Although microbial communities affected by methane seepage have been extensively studied in sediments and seawater, there is a lack of investigation of prokaryotic communities at the surface sediments and bottom seawater. We revealed the effect of methane seepage on co-occurrence networks and ecological niches of prokaryotic communities at the surface sediments and bottom seawater in the Haima cold seep. The results showed that methane seepage could cause the migration of Mn and Ba from the surface sediments to the overlying seawater, altering the elemental distribution at seepage sites (IS) compared with non-seepage sites (NS). Principal component analysis (PCA) showed that methane seepage led to closer distances of bacterial communities between surface sediments and bottom seawater. Co-occurrence networks indicated that methane seepage led to more complex interconnections at the surface sediments and bottom seawater. In summary, methane seepage caused bacterial communities in the surface sediments and bottom seawater to become more abundant and structurally complex. This study provides a comprehensive comparison of microbial profiles at the surface sediments and bottom seawater of cold seeps in the South China Sea (SCS), illustrating the impact of seepage on bacterial community dynamics.

## 1. Introduction

Marine cold seeps are geological structures along continental margins where deeply sourced hydrocarbon-rich fluids are discharged at the seafloor. These seep-derived energy sources, primarily methane, and other hydrocarbons, sustain unique ecosystems [1]. Cold seep fluids provide energy in the form of methane to chemoautotrophic organisms by bacterial action, resulting in thriving biota and carbonates [2,3].

Previous works of literature have conducted abundant research revolving around bacterial action in cold seeps, mainly including biodiversity and co-occurrence. On one side, researchers have compared the archaeal and bacterial diversity in methane seeps with those of other seafloor ecosystems and found that methane seeps harbor unique microbial communities. At the cold seeps, CH_4_ oxidation is associated with communities composed of different functional guilds rather than methanotrophs alone [4]. CH_4_ concentrations at the cold seeps vary intermittently from nanomolar to millimolar concentrations [5,6]. The oxygen concentration in the water will change concurrently as CH_4_ fluctuates, and oxygen availability can determine the composition of the microbial communities involved in CH_4_ oxidation [7,8]. The composition and network properties of microbial communities will change with the presence or absence of methane seepage. Therefore, methane seepage can lead to the creation of special areas at the surface sediments and bottom seawater. On the other side, correlation-based models of microbial co-occurrence networks can reflect the complex interactions within bacterial communities, community assembly, and the overlap of ecological niches. Hence, these models have been applied in numerous studies [9,10]. Differences in ecological niches can directly affect microbial communities and potential functions. For example, studies of benthic ecosystems in sulfate methane transition zones and hydrothermal vents have revealed that methane seepage areas contain unique bacterial taxa [11]. The differences in such taxa reflect important biogeochemical processes [12]. A previous study found that the microbial communities of the surface layer of the seafloor were influenced by water/sediment depth and energy utilization in the sediment [13]. More than 30 cold seeps have been identified in the northern SCS, with only two still active and identified with faunal assemblages [14]. One site is the Haima seep, which was reported for the first time in 2015. Over the past years, most studies on microbial community structure in the Haima cold seep have focused on either seep-affected sediments or the water column [3,15,16,17,18,19]. For example, prokaryotic communities in the bottom water about 50 cm from the seabed at methane seeps with various seepage intensities in Haima, South China Sea were comparatively studied, the abundances of the dominant phyla *Proteobacteria*, *Bacteroidetes*, and *Actinobacteria* differed significantly between non-seepage and the two seepage sites [20].

However, the impacts of methane seepage on co-occurrence networks and ecological niches at the surface sediments and bottom seawater were unclear, remaining a knowledge gap, which is the major focus of this study.

To fill the above knowledge gap, 16S rRNA gene amplification techniques were used to characterize the prokaryotic communities of the surface sediments and bottom seawater environment in different methane seepage areas. The purposes of this study were to answer the following questions: (i) does methane seepage lead to changes in the bacterial community structure; (ii) are there any correlations between environmental factors and bacterial communities; and (iii) how does methane seepage affect the mechanisms of bacterial community assembly?

## 2. Materials and Methods

### 2.1. Samples Collection

**Figure 1:**
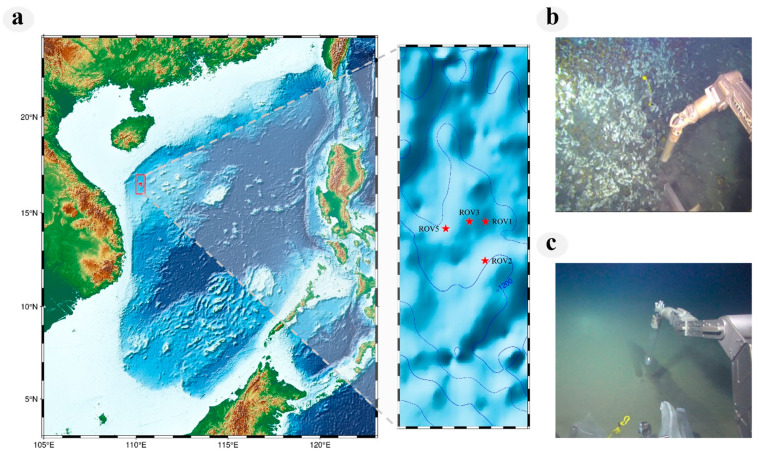
Location and landscapes of the sampling sites. (**a**) Map of the sampling sites with water depth. The box on the right is a zoom-in of the “Haima” area with the locations of four sites. (**b**) Landscapes of the seepage sites (ROV1 and ROV2). (**c**) Landscapes of the non-seepage sites (ROV3 and ROV5).

Samples were collected during an expedition in May 2021 at the Haima cold seep (16°43′ N, 110°28′ E) located in the SCS (Figure 1a). Bottom seawater samples were collected from the water columns above four seepage sites using “Sea-Bird 911” conductivity-temperature-depth (CTD) rosette system distance from the seabed of 160–200 m (Table S1). The surface sediments were collected with gravity corer for depths of 0–6 cm (Table S2) [19]. Four sample sites were selected based on distinct seafloor landscapes, and the sampling sites were divided into two groups. Four sample sites include ROV1, ROV2, ROV3, and ROV5, and the two groups were defined as IS and NS groups. The IS group (ROV1 and ROV2) was identified with biota and continuous seepage (Figure 1b), and the NS group was characterized as (ROV3 and ROV5) outside the active seepage (Figure 1c). One bottle of bottom seawater and one gravity corer of surface sediments were collected in each station. Four bottom seawater and four surface sediment samples were collected from both the NS and IS groups. The distance among the four sites was 1–6.8 km. At each station, 8 L of bottom seawater was immediately filtered through a 0.22 μm polycarbonate membrane (47 mm diameter, Millipore, Billerica, MA, USA) to collect microorganisms. For dissolved CH_4_ measurements in the sample, bottom seawater was collected into 120 mL serum vials, which were bubble-free and poisoned with 200 μL saturated HgCl_2_ immediately. Then, the serum vials were sealed with a butyl rubber stopper with an aluminum cap [21]. The porewater was obtained from the surface sediments using Rhizon samplers [22], and the measurement value of CH_4_ concentration in the porewater represented methane concentration in the surface sediments. When the gravity corers were retrieved to the deck, porewater samples were extracted in less than 15 minutes, and the residual surface sediments and bottom seawater were stored directly at a refrigerator at −80 °C until other parameter tests.

### 2.2. Geochemical Analysis

The Headspace Equilibrium method was used to determine CH_4_ concentrations in seawater using a gas chromatograph [23]. During measurement, 20 mL of the seawater sample was replaced by high-purity nitrogen gas (>99.999%) using two syringes, with one introducing nitrogen gas into the serum vials and the other removing the displaced seawater sample. The serum vials were shaken for 5 min and followed by equilibration for approximately 2 h. CH_4_ concentrations in the porewaters were measured using the head space method and gas chromatography [24,25].

Metal ions and total organic carbon (TOC) concentrations in sediment were represented by the measurement values from porewater extracted from the sediment. The metal ions in the seawater and porewater are diluted with 2% nitric acid and 0.5% hydrochloric acid and measured using an ICP-OES from Thermo Fisher Scientific (Waltham, MA, USA) [26]. The seawater and porewater for the TOC measurement are diluted with ultrapure water to control salinity below 5‰. Finally, the samples were analyzed using a TOC-L total organic carbon analyzer (TOC-L, Shimadzu, Kyoto, Japan) [27]. The above indicators were tested in Guangdong University of Technology.

### 2.3. Deoxyribonucleic Acid Extraction, PCR Amplification, and Sequencing

The microbial DNAs in sediment and bottom seawater were extracted using a power soil DNA extraction kit following the manufacturer’s instructions. The DNA was subjected to quality inspection using a NanoDrop 2000 spectrophotometer (Thermo, ND 2000c, Waltham, MA, USA). Thirty nanograms of qualified genomic DNA of each sample was used for PCR amplification targeting the V3–V4 region of the 16S rRNA gene with primer pairs 341F (5′-CCTACGGGNGGCWGCAG-3′) and 805R (5′-GACTACHVGGGTATCTAATCC-3′) [28]. Primers were tagged with an 8 bp barcode for differentiation of amplicons in the pooled samples multiplexed for Illumina sequencing. The PCR amplification procedure was as follows: initial pre-denaturation at 94 °C for 5 min, a three-step amplification for 30 cycles consisting of denaturation at 94 °C for 30 s, annealing at 55 °C for 45 s, and extension at 72 °C for 1 min, and a final extension at 72 °C for 10 min. The Agencourt AMPure XP magnetic beads (Beckman, Brea, CA, USA) were used to purify the PCR reaction products. An Agilent 2100 Bioanalyzer (Agilent, G2939A, Santa Clara, CA, USA) was used to detect the fragment range and concentration in the library. Eligible libraries were sequenced based on insert size using the HiSeq platform (Illumina, Hiseq2500, San Diego, CA, USA, 2 × 250 bp).

### 2.4. Data Processing and Bioinformation Analysis

Raw reads were processed by the Beijing Genomics Institute (BGI) to generate high-quality sequences (including the removal of adapters, barcodes, primers, and low-quality sequences). The following steps for sequence processing and analysis were conducted mainly with VSEARCH v2.7.0 [29]. The paired-end reads were merged, and this was followed by the discarding of sequences with low quality or shorter than 300 bp [29]. The valid sequences of all samples were clustered using DATA 2 at a 100% concordance level to obtain Amplicon Sequence Varian (ASV) [30]. Taxonomic assignments for the representative sequences of ASVs were determined by aligning the reads to the Silva database (Release 138) [31]. Prior to downstream analysis, all samples are flattened, and the rarified number for all samples is 44,443. The details of sample information of raw sequences are shown in Tables S1 and S2.

The data were analyzed by one-way analysis of variance (ANOVA) for physical and chemical indicators in the environment. SPSS Statistics 25 was used for the analyses.

Alpha diversity indices of bacterial communities in sediment and bottom seawater, including the Shannon index, Chao1 index, Sobs index, and Coverage index, were calculated using the “vegan” and “picante” packages for R Project (version 4.0.5, https://www.r-project.org/ (accessed on 1 October 2023)) [32].

The co-occurrence networks of bacterial communities were constructed at the ASV level. To illustrate the distribution pattern of microbial communities, PCA, redundancy analysis, and Venn diagrams were employed based on “vegan”, “FactoMineR” and “ggvenn” packages for R. The data analysis platform (http://mem.rcees.ac.cn:8080/ (accessed on 1 October 2023)) was used to predict the functionality of bacterial communities through the tool of FAPROTAX. A database of functional annotation of prokaryotic taxa (FAPROTAX) was applied to estimate metabolic and predict biogeochemical cycles (especially the cycle of carbon, nitrogen, and sulfur) or ecological functions [33]. To simplify the co-occurrence network analysis, data filtering was performed beforehand. Then, to unify the analysis, we picked the same ASV number for network analysis. The top 200 ASVs were selected to construct the co-occurrence network. Correlations between species were calculated using Person’s or Spearman’s correlation coefficients (ρ) > 0.9, and *p*-values of the correlation coefficient were corrected using the BH method. Each node represents an ASV, and each edge represents the interaction between two species. The co-occurrence network was visualized using the software Gephi (version 0.9.2). Network-level topological features were calculated through the Gephi platform, including average degree (average connections between nodes), modularity (a measure of whether connections tend to occur within or between modules), graph density (the intensity of connections among nodes, a complete graph has all connected edges with a density of 1), clustering coefficient (overall indication of a node clustering or clumping), and average path length (average graph distance between all node pairs). These parameters were calculated using the R “igraph” package [34].

The niche width function of the R “spaa” package was used to calculate Levins’ niche widths for sediment and water column bacteria [35]. The Raup–Crick dissimilarity index was used to compare community similarity [36,37]. Evaluation of microbial community stability using the AVD index [38]. A neutral community model was used to clarify the potential importance of stochastic processes in microbial community assembly [39].

## 3. Results

### 3.1. Sampling Locations and Environmental Factors

**Figure 2:**
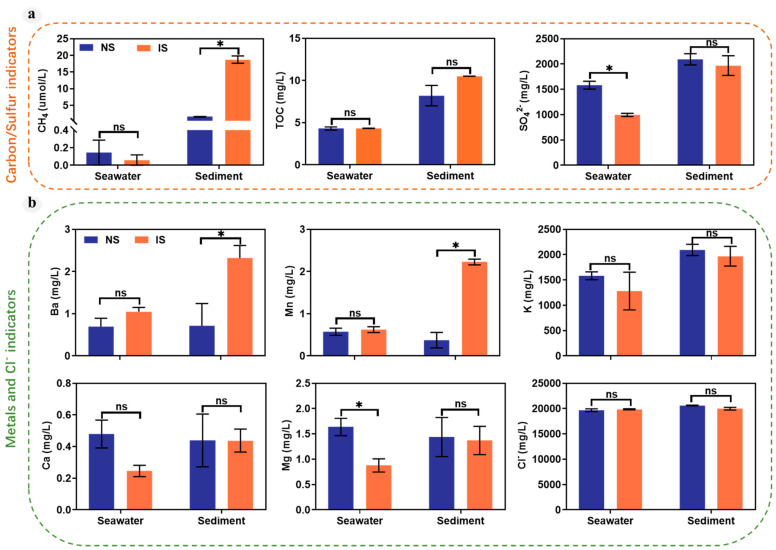
The physicochemical characteristics of seawater and sediment at the surface sediments and bottom seawater. (**a**) CH_4_, TOC and SO_4_^2−^ concentration in seawater and sediment. (**b**) Metals ion and Cl^−^ concentration in seawater and sediment. “ns” means no significant difference between groups; “*” means significant difference between groups (*p* < 0.05; multiple comparison with ANOVA tests).

Concentrations of carbon, sulfur, metals, and other indicators at different stations of the Haima cold seep are shown in Figure 2 and Table S3. The NS Group was located outside the methane seep sites, where the methane concentration in the bottom seawater was 0.05 mg/L, but the biota was not observed on the seafloor (Figure 1c). By contrast, the IS group is located at an active cold seep, where methane fluid, mussels, sea anemones, sea snake tails, and exposed carbonate rocks were observed (Figure 1b). The methane concentration of the surface sediments was 19.50 mg/L.

Apart from methane concentration, the physicochemical properties, including Mn, SO_4_^2−^, Ba, and TOC concentrations at the surface sediments and bottom seawater, exhibited strong lateral variation between different groups. The differences in Mn, Ba, K, Ca, TOC, and Cl^−^ concentrations in seawater of the NS and IS groups were not significant (*p* > 0.05). However, methane seepage in the sediment led to an increase in the Mn and Ba in the IS group. These results suggest that methane seepage has led to the bottom-up migration of elements from the sediment to the bottom seawater.

### 3.2. Microbial Diversity and Structural Differences

**Figure 3:**
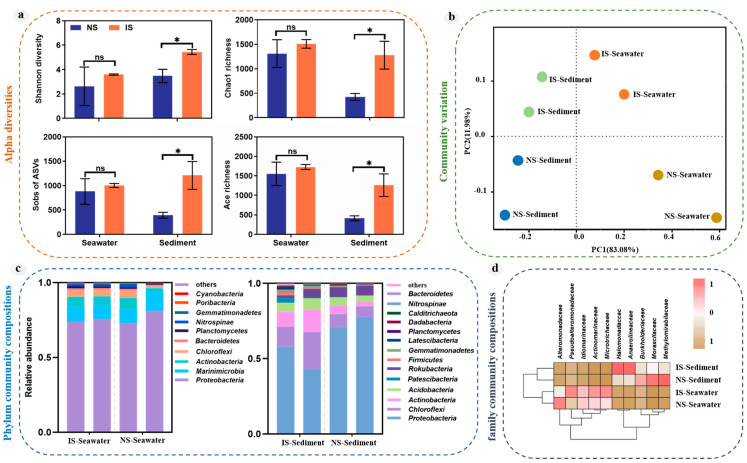
Compositions of bacterial communities at the surface sediments and bottom seawater. (**a**) Microbial alpha diversity in vertical profile. (**b**) PCA of microorganisms at the surface sediments and bottom seawater. (**c**) Graph of differences in microbial composition at the phylum level. (**d**) Graph of differences in microbial composition at the family level. “ns” means no significant difference between groups; “*” means significant difference between groups (*p* < 0.05; multiple comparison with ANOVA tests).

A total of 12,734,072 high-quality reads were acquired after merging and filtering raw data for the eight samples. These reads clustered at 100% sequence similarity, generated 46,186 ASV, and were annotated into 74 phyla, 221 classes, 491 orders, 765 families, 1390 genera, and 1292 species. Rarefaction curves indicated that eight amplicon samples were almost saturated with respect to the number of sequences. Furthermore, the sequencing coverages of eight amplicon samples were above 0.99, indicating that the sequencing depth was sufficient to represent the major diversity of the sequenced samples (Table S4). The results revealed that methane seepage significantly increased bacterial alpha diversity in the sediment but had no significant effect on diversity in the seawater. Hence, methane seepage could result in a richer and more complex bacterial community in the sediment (Figure 3a).

The differences In bacterial community structure were further resolved using PCA analysis. As shown in Figure 3b, the NS and IS groups showed significant differences in bacterial community structure, as methane seepage caused closer distances of bacterial communities between sediment and seawater. This pattern altered the spatial distribution characteristics of the original bacterial communities. The number of shared ASVs between surface sediments and bottom seawater was analyzed using Venn diagrams (Figure S1). The NS group had 67 shared ASV between seawater and sediment, while the IS group of methane seeps had 115 shared ASV between seawater and sediment, indicating that methane seepage caused bacterial species from the sediment to move into the seawater, creating a different habitat compared with the non-seepage sites.

Phyla, accounting for more than 1% of 16S rRNA gene sequences in at least one sample, were selected for analysis. The bacterial communities in the surface sediments and bottom seawater of both the NS and IS groups were dominated by *Proteobacteria*, followed by *Actinobacteria*, and *Marinimicrobia*. Compared with the NS group, methane seepage resulted in a decrease in the abundance of *Proteobacteria* in the surface sediments by approximately 17%, while the abundance of other phyla was increased by 34% (Figure 3c). For example, the surface sediments of the IS showed a higher occurrence of *Chloroflexi*, bacteria that have a very diverse trophic mode, and *Actinobacteria* that are involved in the carbon cycle. The top ten taxa in terms of abundance at the family level were selected to construct a cluster heat map. The samples were clustered into three groups, *Anaerolineaceae* and *Halomonadaceae* as one group and *Burkholderiaceae*, *Moraxellaceae,* and *Methylomirabilaceae* as one group, the others as another group, indicating that methane seepage increased the abundance of *Anaerolineaceae* and *Halomonadaceae* in the surface sediments, and decreased the abundance of *Burkholderiaceae*, *Moraxellaceae*, and *Methylomirabilaceae*, but did not affect the abundance of these species in the bottom seawater (Figure 3d). By contrast, for *Alteromonadaceae*, *Pseudoalteromonadaceae*, *Idiomarinaceae*, *Actinomarinaceae*, and *Microtrichaceae*, methane seepage increased the abundance of bacteria in the bottom seawater in all cases except for *Alteromonadaceae*.

**Figure 4:**
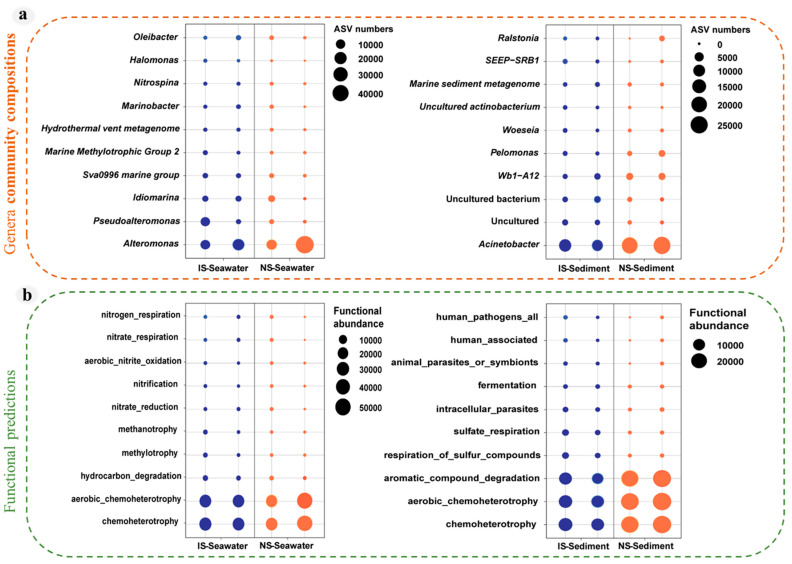
Structures and functions of bacterial communities at the surface sediments and bottom seawater. (**a**) Graph of differences in microbial composition at the genera level. (**b**) Bacterial function predictions based on the FAPROTAX tool.

According to the heat map, the relative abundance of the top 10 species genera was analyzed, and we found that methane seepage caused a 25% and 21% decrease in the relative abundance of *Alteromonas* in bottom seawater and *Acinetobacter* in surface sediments (Figure 4a). To validate this conclusion, FAPROTAX was used to predict whether methane leakage would cause changes in community function. The study showed that bacterial functional groups related to chemosynthesis were significantly decreased in surface sediments and bottom seawater (Figure 4b).

**Figure 5:**
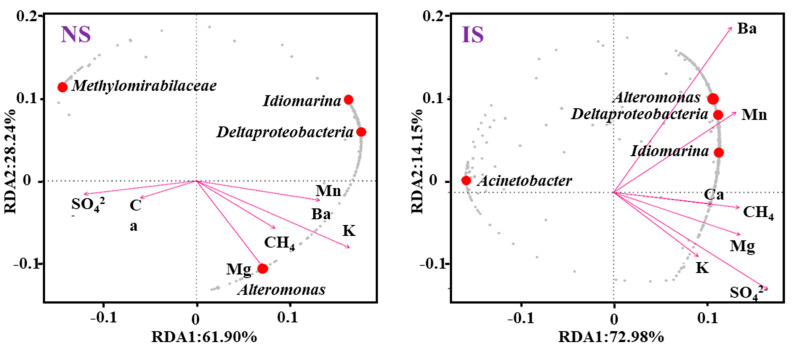
RDA of bacterial community structure and environmental factors.

Microbial community structure is significantly influenced by the environment, and the results of redundancy analysis (RDA) can reflect the relationship between community structure and environmental factors and identify the important environmental drivers affecting microbial distribution. The correlations between bacterial community structure and environmental factors in the NS and IS groups are shown in Figure 5. The RDA results showed that SO_4_^2–^ and Mn were the most significant drivers of bacterial community structure in the NS group (Figure 5). The order of influence of environmental factors on the bacterial community structure of the IS group in the methane seepage zone was CH_4_ > Ca > Mg, with CH_4_ being the most significant driver of bacterial community structure in the IS group (*p* < 0.05) (Figure 5). The sum of RDA1 and RDA2 explained 90.14% and 87.13% of the changes in bacterial community structure in the sediment and seawater, respectively. Thus, CH_4_ was an important environmental factor in the altered bacterial community structure.

### 3.3. Co-Occurrence Networks and Stability of Bacterial Communities

**Figure 6:**
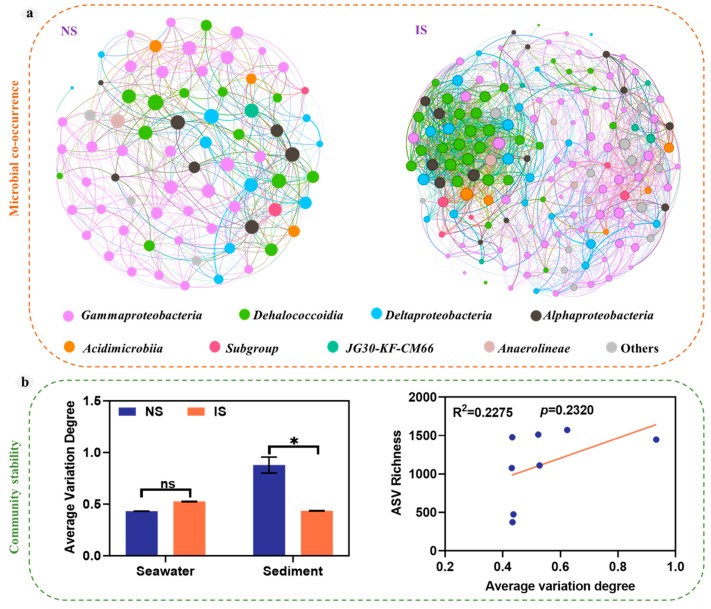
Co-occurrence networks and stability of bacterial communities in the NS and IS groups. (**a**) The nodes are colored based on the phylum level of prokaryotic microorganisms. A connection indicates a strong (Spearman’s ρ > 0.9) and significant (*p* < 0.01) correlation. The size of each node is proportional to the degree of ASV. (**b**) The relationship between bacterial ASV richness and average variation degree (AVD) of assembled bacterial communities. “ns” means no significant difference between groups; “*” means the significant difference between groups (*p* < 0.05; multiple comparisons using ANOVA tests).

The co-occurrence networks of inter-group bacteria were constructed at the ASV level (Figure 6a), and the corresponding network topology parameters are listed in Table 1. Methane seepage led to significant differences in the co-occurrence network of the bacterial community between NS and IS groups. The NS group bacterial occurrence network for non-methane seepage consisted of 198 nodes and 1307 edges, and the IS group bacterial occurrence network for methane seepage consisted of 198 nodes and 2374 edges (Table 1). These results indicated that methane seepage resulted in closer interconnections among species. In addition, the mean degree of IS group nodes was significantly higher than in the NS group, while modularity was lower than in the NS group, indicating greater stability of the co-occurrence networks of bacteria due to methane seepage. Furthermore, we analyzed community stability and found that methane leakage reduced community stability in sediments, while community stability was linearly correlated with ASV richness (Figure 6b).

### 3.4. Process Differences in Bacterial Community Construction

**Figure 7:**
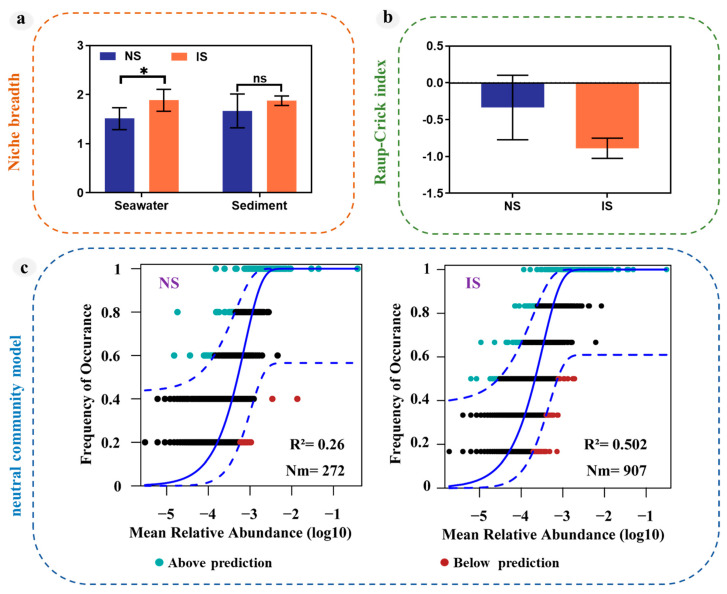
Mechanisms of bacterial assembly at the surface sediments and bottom seawater. (**a**) Niche breadth of bacterial communities at the surface sediments and bottom seawater. (**b**) Assessment of community structure based on the Raup–Crick index. (**c**) Assessment of the relative importance of deterministic and stochastic processes in community assembly based on the neutral community models. The solid blue line represents the best-fit value of the neutral community models, the dashed blue line represents the 95% confidence interval of the model, ASVs with a higher or lower frequency than predicted by the neutral community models are shown in different colours. “ns” means no significant difference between groups; “*” means significant difference between groups (*p* < 0.05; multiple comparison with ANOVA tests).

For further clarification of the effect of methane seepage on the niche breadth of bacterial communities, the Levins and Shannon formulas were applied to calculate the top ten bacterial species. The IS group of the methane seepage showed a significantly increased niche breadth of bacterial communities in the water column (Figure 7a) that affected the survival of major bacteria, such as *Alteromonadaceae*, *Deltaproteobacteria*, *Microtrichales*, indicating that methane leakage causes some bacteria to be lacking key nutrient sources, forcing them to need to adapt to the wider environment. We calculated the mechanism of bacterial community assembly through the Raup–Crick index (Figure 7b), and the results showed that the IS group of methane seepage had a more similar bacterial community. It was clear that methane was present as a stronger ecological filter in this environment. A tighter constraint was placed on the bacterial community within this environment. Moreover, neutral community models used for analysis, we found that methane seepage leads to community construction influenced more by stochastic processes and increases the dispersal of species (Figure 7c).

## 4. Discussion

### 4.1. Effects of Methane Seepage on the Environment and Bacterial Communities

The migration of methane fluids in sediment and subsequent release into bottom waters typically result in the formation of venting structures [40]. These so-called “cold” seeps are particularly common at margins and hence could play an important role in the exchange processes between seawater and sediment. The distribution patterns of elements provide useful information on both fluid sources and the origin of particulate phases at submarine cold seeps [41,42]. The emission of methane-rich fluids at the seafloor is typically associated with high particulate contents, and the mixing of anoxic fluids with oxygen-rich bottom waters leads to the precipitation of Mn oxyhydroxides in methane plumes. For example, Mn enrichment in bottom waters overlying active seepage sites is diagnostic of sediment resuspension due to the emission of gas-rich fluids at submarine vents [41,43,44].

In addition to the methane concentration as the key environmental factor, we also found that Mn and Ba concentrations were correlated with methane seepage (*p* < 0.05) (Figure 2). This may be due to the removal of large amounts of dissolved Mn from the pore water during the upward migration of the methane fluid, resulting in increased elemental Mn in the surface sediments. In addition, precipitation of authigenic carbonate minerals (e.g., aragonite, high-Mg calcite) commonly takes place in anoxic sub-surface sediments or in bottom waters in relation to the anaerobic oxidation of methane [45]. The high rare earth element contents determined in methane-derived carbonates suggest that carbonate precipitation represents an important sink for rare earth elements in marine sediments [46,47,48]. This has been recently confirmed in a study reporting pore water rare earth elements data at Hydrate Ridge (NE Pacific Ocean), which showed that rare earth elements were almost completely depleted at the sulfate–methane interface [49]. Intense carbonate precipitation can affect the local distribution of rare earth elements, where both AOM and carbonate formation occur a few centimeters below the seafloor. This ultimately results in rare earth elements not entering the water column. Cold seep vents are also a source of Ba in the ocean. The estimate for the cold seeps contribution of Ba is between 1/10 and 1/3 of the total input of Ba to the ocean, with concentrations in these fluids ranging from 10 to 40 μmol/kg [42]. The major carrier of particulate Ba in the water column is the mineral barite [40].

Methane seepage can select specific and highly adapted colonizers from a reservoir of organisms that are dispersed to the surface of the seafloor by bottom currents or moving sediments, where long-term selection of the environment leads to the possible formation of different bacterial communities in methane seeps [50,51]. Methane seepage has been shown to vary intermittently from nanomolar to millimolar; for example, in the 2010 study of the oil spill that occurred in the Gulf of Mexico, the main bacterial taxa affected were members of *Gammaproteobacteria*, *Alphaproteobacteria*, and *Bacteroidia* [52,53], and there were significant differences for the dominant microbial lineages between the different seepage events [54,55]. We observed that methane seepage increased the abundance of *Anaerolineaceae* and *Halomonadaceae* in the surface sediments and decreased the abundance of *Burkholderiaceae*, *Moraxellaceae,* and *Methylomirabilaceae*, but did not affect the abundance of these species in the bottom seawater (Figure 3d). In addition, methane seepage significantly increased bacterial alpha diversity in the sediment but had no significant effect on the seawater (Figure 3a). Therefore, methane seepage resulted in a richer and more complex bacterial community in the sediment compared with seawater, altering the spatial distribution characteristics of the original bacterial community.

### 4.2. Effects of Methane Seepage on Bacterial Community Construction

Co-occurrence networks further revealed variable microbial interactions across methane seepage regions. As shown by node-level and network-level topological characteristics, modules were more likely to have independent functional ecological niches [56]. This difference could be attributed to the more diverse and abundant bacterial communities in the sediments being disturbed by methane seepage, resulting in more complex interactions (symbiosis, competition, or predation) within bacterial communities and a more complex network structure [57]. Previous research has also shown that microorganisms form close associations in stable environments [58]. In co-occurrence networks, nodes with high interconnectivity with others are considered “hubs” of microbial communities, e.g., *Acinetobacter* and *Woeseia*; they are mainly based on aerobic respiration. These hubs drive the evolution of microbial community composition and function [59]. In this study, methane seepage resulted in closer interconnections among species (Figure 6a). In addition, the mean degree of IS group nodes was significantly higher than in the NS group, while modularity was lower than in the NS group, indicating higher stability of the co-occurrence networks of bacteria due to methane seepage (Table 1).

The ecological niche of a species determines the functions it performs in an ecosystem, while niche width defines the species’ resource-use capacity and represents a key factor influencing species diversity and community structure [60]. Cold seeps are recognized as important ecosystems that are key to biological production in the deep sea [61,62]. In these environments, fluids enriched in methane, sulfide, and other reduced compounds are emitted from the seafloor and utilized by free-living and symbiotic microbes (e.g., methanotrophic and/or sulfur-oxidizing bacteria) [63]. Seep assemblages are often characterized by the presence of large chemosymbiotic megafauna (e.g., mytilid bivalves, vesicomyid clams, vestimentiferan and frenulate polychaetes, gastropods, and sponges) and surface bacterial mats [64]. Microbial production by bacteria and archaea at cold seeps forms the basis of complex benthic food webs characterized by diverse energy pathways and carbon sources [65,66]. Therefore, compared with the non-methane seepage NS group, we observed that methane seepage significantly increased the niche breadth of bacteria in the bottom water column, as competition between bacterial species was more intense and the wider niche allowed for a more adaptive bacterial community (Figure 7a). Therefore, methane seepage changes trophic structure within the original habitat, causing bacteria to readapt to the new environment and expand their ecological niche.

Deterministic or stochastic processes of assembly determine the composition and diversity of microbial communities and their function. Dispersal is a key factor affecting the regional species pool and its related community composition. A high dispersion rate can homogenize the community composition, resulting in little change or turnover. For example, stochastic processes dominated the community assembly of the water column [67]. This study also analyzed the relative importance of deterministic and stochastic processes in the assembly of communities in methane seepage regions. The results indicated a high degree of similarity within the IS group for methane seepage, with methane as a strict ecological filter at the surface sediments and bottom seawater and stochastic processes playing a dominant role (Figure 7c).

## 5. Conclusions

Currently, there is a lack of investigation of prokaryotic communities at the surface sediments and bottom seawater. The present study explored the impacts of methane seepage on bacterial communities in surface sediments and bottom seawater in Haima cold seep areas. The main conclusions are as follows:

(1) Methane seepage contributed to the migration of some elements in the sediment, altering the original elemental distribution patterns;
(2) Methane seepage led to an increase in the similarity of bacterial communities in the bottom water and surface sediments and increased the abundance and diversity of bacteria in the sediment;
(3) The richer and more complex bacterial community in the sediment, with the stochastic processes, played a dominant role in the bacterial community assembly.

## Figures and Tables

**Table 1:** Topological properties of bacterial co-occurrence networks.

Group	Nodes	Edge	Mean Clustering Coefficient	Mean Degree	Mean Path Length	Modularity
NS	198	1307	0.587	13.202	3.852	0.52
IS	199	2374	0.496	23.859	2.886	0.509

## Data Availability

All Illumina Miseq raw sequence data without spike-in sequences were deposited in the NCBI Sequence Read Archive (accession code PRJNA909048 and PRJNA845826).

## Supplementary Materials

The following supporting information can be downloaded at https://www.mdpi.com/article/10.3390/microorganisms11123001/s1, Figure S1: Venn diagrams of common and specific ASV at different sites; Table S1: The details of seawater sample information of raw sequences deposited at the NCBI; Table S2: The details of sediment sample information of raw sequences deposited at the NCBI. Depth, the distance relative to the top of sediment core; Table S3: Geochemical characteristics of the seawater and sediment above the Haima cold seep across vertical profile, CH_4_ (µmol/L), Mn (mg/L), Ba (mg/L), Mg (mg/L), Ca (mg/L), TOC (mg/L), SO_4_^2−^ (mg/L), K (mg/L); Table S4: α-diversities of the prokaryotic communities in the seawater and sediment above the Haima cold seep.
