# Supplementary material for: Differences in Bacterial Co-Occurrence Networks and Ecological Niches at the Surface Sediments and Bottom Seawater in the Haima Cold Seep
Source: Microorganisms. 2023 Dec 18;11(12):3001. doi: 10.3390/microorganisms11123001 (PMC10745737; doi:10.3390/microorganisms11123001)
Supplement: Supplementary file 1 [file microorganisms-11-03001-s001.zip › microorganisms-2696120-supplementary.pdf]

Differences in Bacterial Co-Occurrence Networks and Ecological Niches at the Surface Sediments and Bottom Seawater in the Haima Cold Seep

Song Zhong [1,2,†], Jingchun Feng [1,2,3,*], Jie Kong [2,3,†], Yongji Huang [2,3], Xiao Chen [2,3] and Si Zhang [2,3]

[1] Guangdong Provincial Key Laboratory of Water Quality Improvement and Ecological Restoration for Watersheds, Institute of Environmental and Ecological Engineering, Guangdong University of Technology, Guangzhou 510006, China; 1112124003@mail2.gdut.edu.cn

[2] Research Centre of Ecology & Environment for Coastal Area and Deep Sea, Guangdong University of Technology, Guangzhou 510006, China; kongjie@gmlab.ac.cn (J.K.); yjhuang@scsio.ac.cn (Y.H.); chen_xiao@gmlab.ac.an (X.C.); zhsimd@scsio.ac.cn (S.Z.)

[3] Southern Marine Science and Engineering Guangdong Laboratory (Guangzhou), Guangzhou 511458, China

* Correspondence: fengjc@gdut.edu.cn

† These authors contributed equally to this work.

List of supplemental information

Table S1. The details of seawater sample information of raw sequences deposited at the NCBI.

Table S2. The details of sediment sample information of raw sequences deposited at the NCBI. Depth, the distance relative to the top of sediment core.

Table S3. Geochemical characteristics of the seawater and sediment above the Haima cold seep across vertical profile. CH_4 (μmol/L), Mn (mg/L), Ba (mg/L), Mg (mg/L), Ca (mg/L), TOC (mg/L), SO_4^{2-}(mg/L), K(mg/L).

Table S4. α-diversities of the prokaryotic communities in the seawater and sediment above the Haima cold seep.

Figure S1. Venn diagrams of common and specific ASV at different sites.

Table S1. The details of seawater sample information of raw sequences deposited at the NCBI.

Bioproject ID	*SRA accession number*	Site	Water depth
PRJNA909048	SRX18543112	ROV1	1350 m
PRJNA909048	SRX18543114	ROV2	1350 m
PRJNA909048	SRX18543125	ROV3	1350 m
PRJNA909048	SRX18543147	ROV5	1350 m

Table S2. The details of sediment sample information of raw sequences deposited at the NCBI. Depth, the distance relative to the top of sediment core.

Bioproject ID	*SRA accession number*	Site	Depth
PRJNA845826	SRX15602217	ROV1	5cm
PRJNA845826	SRX15601990	ROV2	5cm
PRJNA845826	SRX15602176	ROV3	6cm
PRJNA845826	SRX15602145	ROV5	5cm

Table S3. Geochemical characteristics of the seawater and sediment above the Haima cold seep across vertical profile. CH_4 (μmol/L), Mn (mg/L), Ba (mg/L), Mg (mg/L), Ca (mg/L), TOC (mg/L), SO_4^{2-}(mg/L), K(mg/L).

Site	Type	CH_4	Mn	Ba	Mg	Ca
ROV1	seawater	0.0136	0.573	1.119	0.785	0.22
	sediment	19.499	2.177	2.11	1.567	0.489
ROV2	seawater	0.1001	0.671	0.973	0.969	0.271
	sediment	17.947	2.273	2.53	1.173	0.386
ROV3	seawater	0.2443	0.632	0.834	1.516	0.417
	sediment	1.6299	0.242	0.34	1.164	0.321
ROV5	seawater	0.0433	0.5104	0.555	1.757	0.541
	sediment	1.5142	0.502	1.086	1.71	0.557

Table S3. Geochemical characteristics of the seawater and sediment above the Haima cold seep across vertical profile. CH_4 (μmol/L), Mn (mg/L), Ba (mg/L), Mg (mg/L), Ca (mg/L), TOC (mg/L), SO_4^{2-}(mg/L), K(mg/L), Cl^-(mg/L) (continued).

Site	Type	TOC	SO_4^{2-}	K	Cl^-
ROV1	seawater	4.336	1017	261	19791
	sediment	10.5	1831	636	19801
ROV2	seawater	4.311	972	295	19932
	sediment	10.51	2106	352	20158
ROV3	seawater	4.179	1528	427	19495
	sediment	9.068	2014	746	20578
ROV5	seawater	4.442	1637	580	19871
	sediment	7.35	2171	580	20656

Table S4. α-diversities of the prokaryotic communities in the seawater and sediment above the Haima cold seep.

Site	Type	Sobs of ASV	Chao1 richness	Ace richness	Shannon diversity	Coverage
ROV1	seawater	975	1447	1684	4	0.99
	sediment	1412	1478	1466	6	0.99
ROV2	seawater	1032	1572	1772	4	0.99
	sediment	1008	1077	1057	5	0.99
ROV3	seawater	1066	1512	1766	4	0.99
	sediment	351	373	371	3	0.99

Table S4. α-diversities of the prokaryotic communities in the seawater and sediment above the Haima cold seep (continued).

Site	Type	Sobs of ASV	Chao1 richness	Ace richness	Shannon diversity	Coverage
ROV5	seawater	694	1111	1340	2	0.99
	sediment	436	474	458	4	0.99

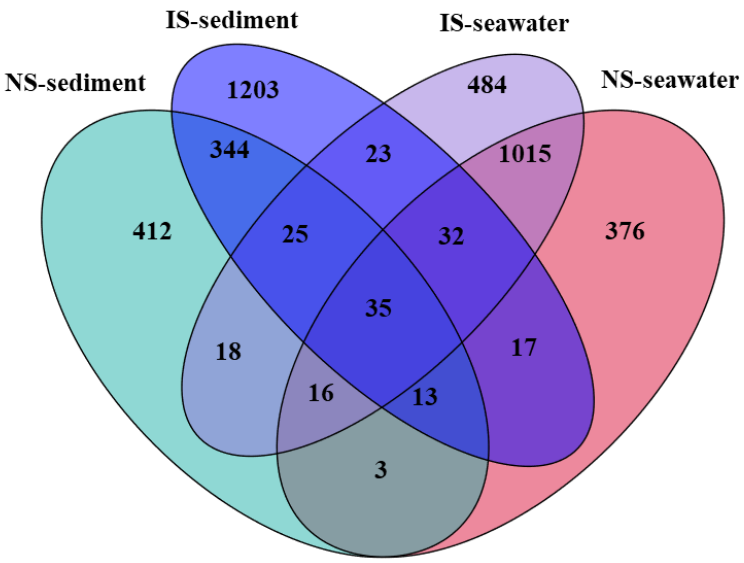

Figure S1. Venn diagrams of common and specific ASV at different sites.
